# An international survey of patients with cervical dystonia

**DOI:** 10.1007/s00415-014-7586-2

**Published:** 2015-01-22

**Authors:** Cynthia Comella, Kailash Bhatia

**Affiliations:** 1 Department of Neurological Sciences, The Movement Disorders Center at Rush University Medical Center, 1725 West Harrison St., Suite 755, Chicago, IL 60612 USA; 2 Institute of Neurology, University College London, London, UK

**Keywords:** Cervical dystonia, Patient, Survey, Treatment, Satisfaction, Botulinum toxin

## Abstract

This was an international survey undertaken to assess cervical dystonia (CD) patients own perceptions of their illness and its management. A total of 1,071 self-identified respondents with CD in 38 countries completed the online survey between March and December 2012. The mean time since diagnosis was 9.6 years and over half (54 %) of patients surveyed were not diagnosed in the first year. When asked how the symptoms of CD affected them, two-thirds (66 %) of patients reported they experienced a lot of pain, and 61 % said that they suffered depression and mood alterations; only 7 % reported no impact on their lives. Despite problems with the diagnosis, almost 70 % of respondents reported being satisfied with the overall relationship with their doctor. Patient treatment expectations were high, with 63 % expecting freedom from spasms and 62 % expecting freedom from pain. Over half (53 %) expected to be able to return to a normal routine (53 %). The most common treatment reported was botulinum toxin (BoNT) (86 %), followed by oral medication (58 %) and physiotherapy/physical therapy (37 %). Among patients treated on BoNT, 56 % were fairly/very satisfied, 25 % were fairly/very dissatisfied and 20 % were neither satisfied nor dissatisfied with the outcome. In conclusion, this international survey highlights the broad impact of CD on several aspects of patient life. Taken overall, the survey suggests that that patients need to be better informed about their condition, treatments available and the limitations of those treatments. It may be that realistically managing patient expectations of treatment would reduce the dissatisfaction of some patients.

## Introduction

Cervical dystonia (CD) is a chronic neurologic condition primarily characterized by the twisting or turning of the neck, and/or displacement of the head. Disability with functional impairment, pain and embarrassment with social withdrawal are also frequent features of the disorder and several studies have shown that CD can have important impact on the patient quality of life [1–3]. The average age of CD onset is around 41 years [4, 5]; consequently, many patients are working and have young families when they are diagnosed [6]. The impact on daily life is significant and it has been reported that people with CD retire at least 10 years earlier than the general population due to their condition [7].

Current treatment guidelines recommend injections of botulinum toxin (BoNT) as first-line treatment for primary CD [8]. The efficacy and safety of BoNT in the management of CD are well established [9] and several studies have shown that treatment with BoNT improves quality of life in CD [10–12]. However, it has been reported that patient satisfaction can be lower than expected for such an effective treatment [13, 14] and other studies have shown that psychosocial factors (e.g., presence of depression, social support) are also important considerations in patient management [1, 11–15]. The aim of this international online survey was to assess patient views and perceptions on the impact of their illness and its treatment,

## Methods

### Survey design

This international online survey was conducted between March and December 2012. The structure and contents of the survey were designed in collaboration with Dystonia Europe and the Dystonia Medical Research Foundation. The study was supported by Ipsen Pharma.

The survey was designed to be self-report by the patients; all online survey responses were purely voluntary and as such no ethics approval was sought. It was first designed in English, and then translated into French, German and Spanish; native speakers verified all translations. The survey was hosted online and included 4 demographic screening questions and 38 disease-related questions grouped into categories (“Appendix”). All of the questions were multiple choice and 13 included a free entry format as one of the options. Survey responses were anonymous. The survey was designed to take approximately 15 min to complete; however, there was no set time limit for completion.

### Survey participants

People with a diagnosis of CD were invited to participate in the survey by two patient associations: Dystonia Europe (including their local patient association members) and the Dystonia Medical Research Foundation, via a social media campaign. The patient associations hosted links to the patient survey online, and CD patients were also directed to the survey through patient society e-mail communications, newsletters, meetings, and social networking sites (Facebook and Twitter). Other than having a diagnosis of CD (self-reported), there were no formal inclusion or exclusion criteria for participating in this survey.

### Data analysis

Descriptive statistics were used to summarize all survey data collected in this study.

## Results

### Sample

Over the 10 months that the First International CD Patient Survey was available online, 1,071 respondents with self-identified CD completed the survey. Participants were from five continents (38 countries: Austria, Australia, Belgium, Brazil, Canada, Croatia, Czech Republic, Denmark, El Salvador, Finland, France, French Guinea, Germany, Guyana, Iceland, Ireland, Israel, Italy, Kenya, Mexico, Netherlands, New Zealand, Norway, Oman, Philippines, Poland, Portugal, Romania, Russia, Slovak Republic, Spain, South Africa, Sweden, Switzerland, Taiwan, UK, USA, Vietnam) The mean (±SD) age of respondents was 53.2 ± 11.9 years and the majority were female (Table 1). In terms of employment, 42 % of respondents were employed [full time (24 %), part time (12 %), self-employed (6 %)], 22 % were retired and 9 % were not currently employed or were full-time students. Twenty-six percent responded that they were unable to work/disabled.

**Table 1:** Demographic and clinical characteristics

Characteristic	Statistic
Female; *n* (%)	813 (76)
Age (years); mean ± SD	53.2 ± 11.9
18–24	12 (1 %)
35–44	59 (6 %)
45–54	346 (32 %)
55–64	281 (26 %)
≥65	210 (20 %)
Employment status; *n* (%)	
Full time (>30 h/week)	256 (24 %)
Self-employed	62 (6 %)
Full-time student	131 (12 %)
Retired	239 (22 %)
Not able to work/disabled	283 (26 %)
Not currently employed	89 (8 %)
Time since diagnosis (years); mean ± SD	9.6 ± 6.3
Presence of another movement disorder; *n* (%)	393 (37)
Blepharospasm	84 (8)
Other dystonias (non-specified)	335 (31)
Hemifacial spasm	35 (3)

### Impact of CD on daily living

**Fig. 1:**
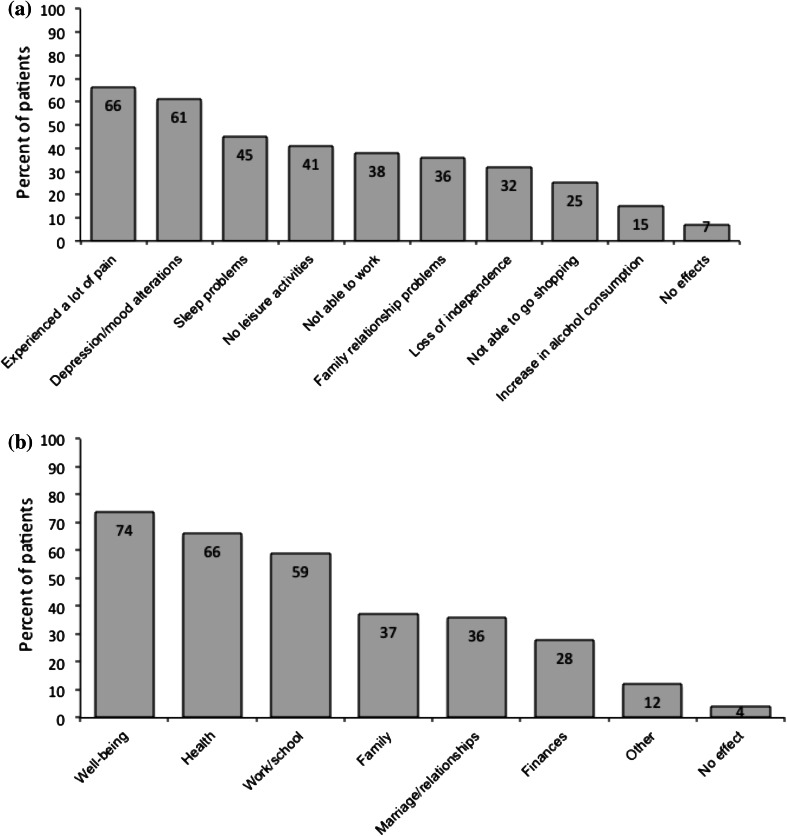
Impact of CD on daily life **a** individual problems **b** overall areas of life. Base = 1,071 patients in survey. Patients were asked: after your first symptoms began **a** how did they affect and change your life? **b** Which of the following areas of life were affected when your symptoms were at their worst?

When asked how the symptoms of CD affected them after their symptoms first began, two-thirds (66 %) of patients reported they experienced pain, and 61 % said that they suffered depression and mood alterations; only 7 % of respondents reported no impact on their lives (Fig. 1a). When asked to consider the areas of life most affected when symptoms were at their worst, a majority of patients reported a negative impact on general well-being, health and work/school life (Fig. 1b). Overall, 12 % of patients reported an effect on ‘other’ life areas, which included confidence and self-esteem, isolation, sports and exercise.

### Diagnostic issues

Late diagnoses and misdiagnoses were frequently reported in the survey. Less than half (46 %) of patients were diagnosed within a year of developing symptoms, 29 % were diagnosed within 1–5 years, and 25 % of patients reported that it took at least 5 years for them to receive their diagnosis. In total, 66 % of patients reported being misdiagnosed. The most frequent misdiagnoses were: psychological illness or stress disorder (37 %), cervical muscle strain (23 %) and tremor (15 %). Overall, 19 % of patients reported ‘other’ diagnoses, which included Parkinson’s disease, essential tremor, multiple sclerosis, hypochondria, cervical arthritis, scoliosis and whiplash.

After diagnosis, the majority (76 %) of patients looked for more information on the Internet. Other sources of information included patient associations (31 %), printed information at the doctors office (23 %), and primary care doctor (20 %). Responses were generally consistent across countries, except in Spain where 69 % consulted a patient association for more information and only 50 % searched the Internet.

When first discussing CD with their neurologist/movement disorder specialist, only 53 % of patients were satisfied with the information they received about their condition. The main reasons given for dissatisfaction were: lack of materials provided (72 %), not enough time with physician to explain the diagnosis (52 %) and feeling overwhelmed by the diagnosis (14 %). Of those who reported dissatisfaction with the information provided (*n* = 501), over half said they wanted more time with their doctor to better understand the diagnosis (*n* = 259, 52 %). When these patients were questioned further about how much time they thought was required to discuss a new diagnosis, most (52 %) thought that *a* > 30 min consultation was necessary.

### Therapeutic relationship and self-management

**Fig. 2:**
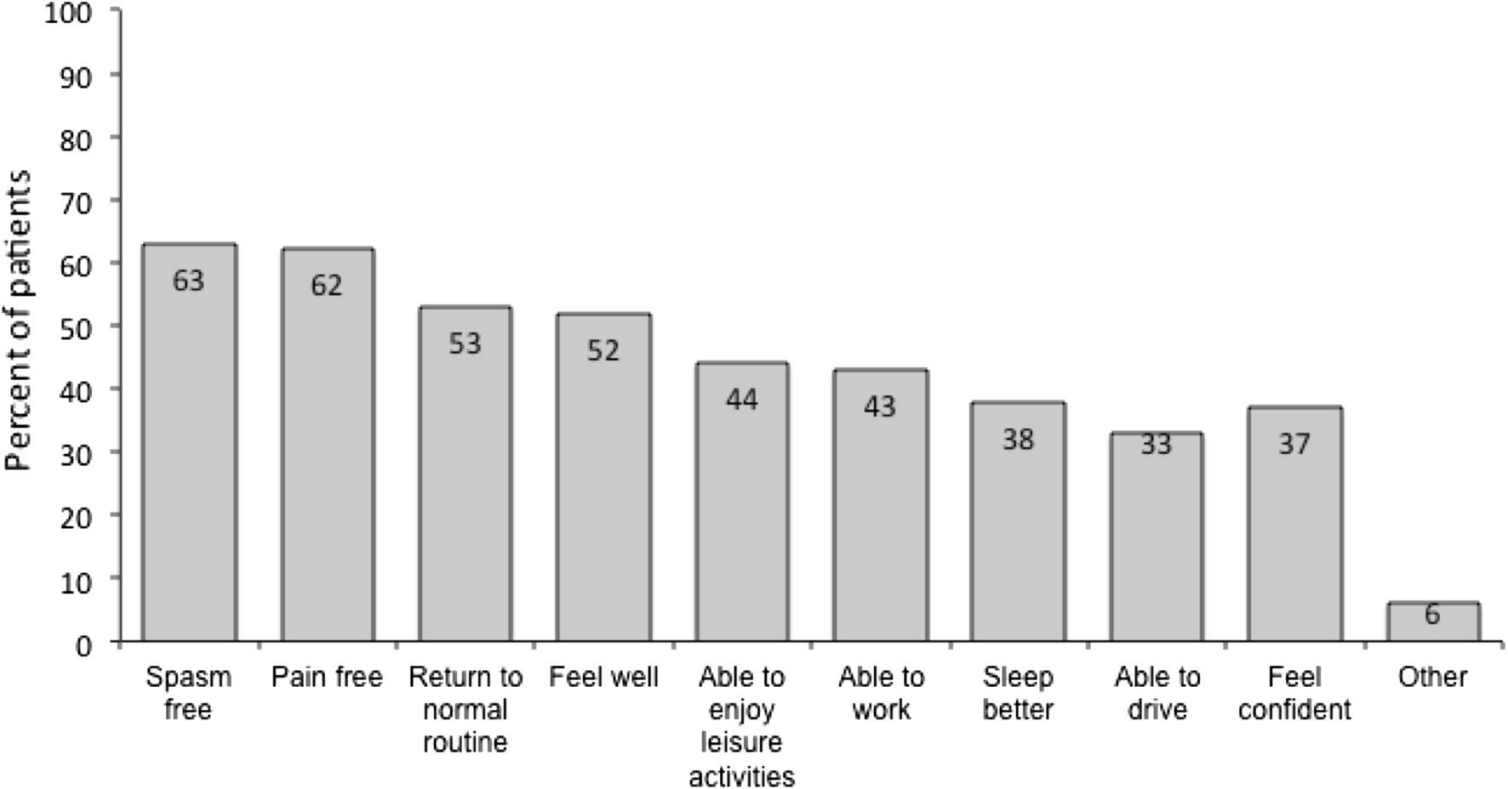
Expectations of treatment following discussion with a doctor. Base = 772 patients who had discussed expectations regarding treatment. Patients were asked: after discussing the different treatment options with your doctor, what were your expectations regarding your overall treatment plan?

Despite problems with the diagnosis, almost 70 % of survey respondents reported being satisfied with the overall relationship with their doctor. During appointments with their physicians, 72 % of patients said that they had discussed treatment expectations. Patient expectations were high, with 63 % expecting freedom from spasms and 62 % expecting freedom from pain. Over half (53 %) expected to be able to return to a normal routine (53 %) (Fig. 2).

With regards to self-management, most patients in the survey (72 %) had contacted a patient association during the course of their disease and of these 86 % would recommend patient societies to other patients. In addition, the majority of patients (91 %) reported trying practical, self-managed interventions to improve their condition. The four most successful interventions were reported as: general rest (41 %), physiotherapy (28 %), light exercise (25 %) and massages (23 %). Other interventions deemed successful included: stress reduction training, Pilates/yoga, gardening, creative endeavors such as painting or singing, reflexology and using a foam or water pillow. As a result of these practical interventions, patients felt an improvement in symptoms (41 %), mood improvement (35 %) and gained confidence (27 %), but some patients (32 %) experienced no change.

### Medical treatment of CD

Most patients included in the survey (86 %) had been treated with BoNT. Other treatments chosen or prescribed (at any time) included: oral medication (53 %), physiotherapy (31 %), psychological support (11 %) and surgery/deep brain stimulation (9 %). Only 21 patients (2 %) did not receive any treatment. As a result of these medical intervention(s), most patients (64 %) reported an improvement in symptoms. Other reported benefits included mood improvement (39 %), gained confidence (31 %), regained independence (22 %), less anxiety when driving (21 %) and ability to return to work (17 %). By contrast, 16 % of patients reported that they had not experienced any improvements. With regards to treatment satisfaction with their CD treatment, 51 % of patients reported that they were fairly or completely satisfied, 21 % were neither satisfied nor dissatisfied, and 28 % reported that they were fairly or completely dissatisfied with their treatment outcomes.

**Fig. 3:**
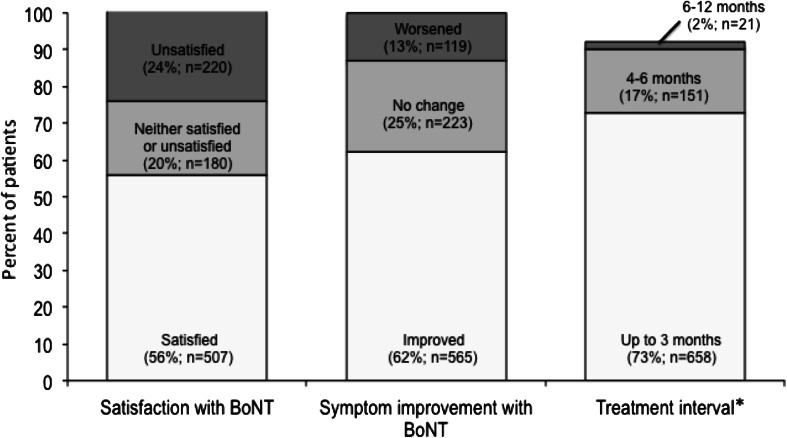
Patient experience with botulinum toxin. Base = 907 patients who had received treatment with botulinum toxin. *Asterisk* a total of 77 patients (8 %) reported they received their BoNT injection when required. Patients were asked: 28. How do you rate the overall satisfaction of botulinum toxin treatment? Since receiving treatment with botulinum toxin have your symptoms improved, worsened or stayed the same? Based on your experience with botulinum toxin treatment, how long do you have to wait between treatments?

Of the patients who reported receiving BoNT (*n* = 907), the majority of patients (80 %) reported that they had discussed the potential benefits and side effects of treatment with their doctor. Most (73 %) patients reported an injection interval of 3 months between treatments, 17 % reported an interval of 4–6 months, and 2 % reported an interval of 7–12 months (Fig. 3). In addition, a further 8 % reported they received their BoNT when required. Since receiving treatment with BoNT (*n* = 907); 62 % of patients reported symptom improvement, 25 % reported no improvement and 13 % reported a worsening of symptoms. Considering their BoNT treatment, over half (56 %) of patients reported that they were completely or fairly satisfied with their treatment, 20 % were neither satisfied nor dissatisfied, and almost one quarter (24 %) were completely or fairly dissatisfied (Fig. 3).

**Fig. 4:**
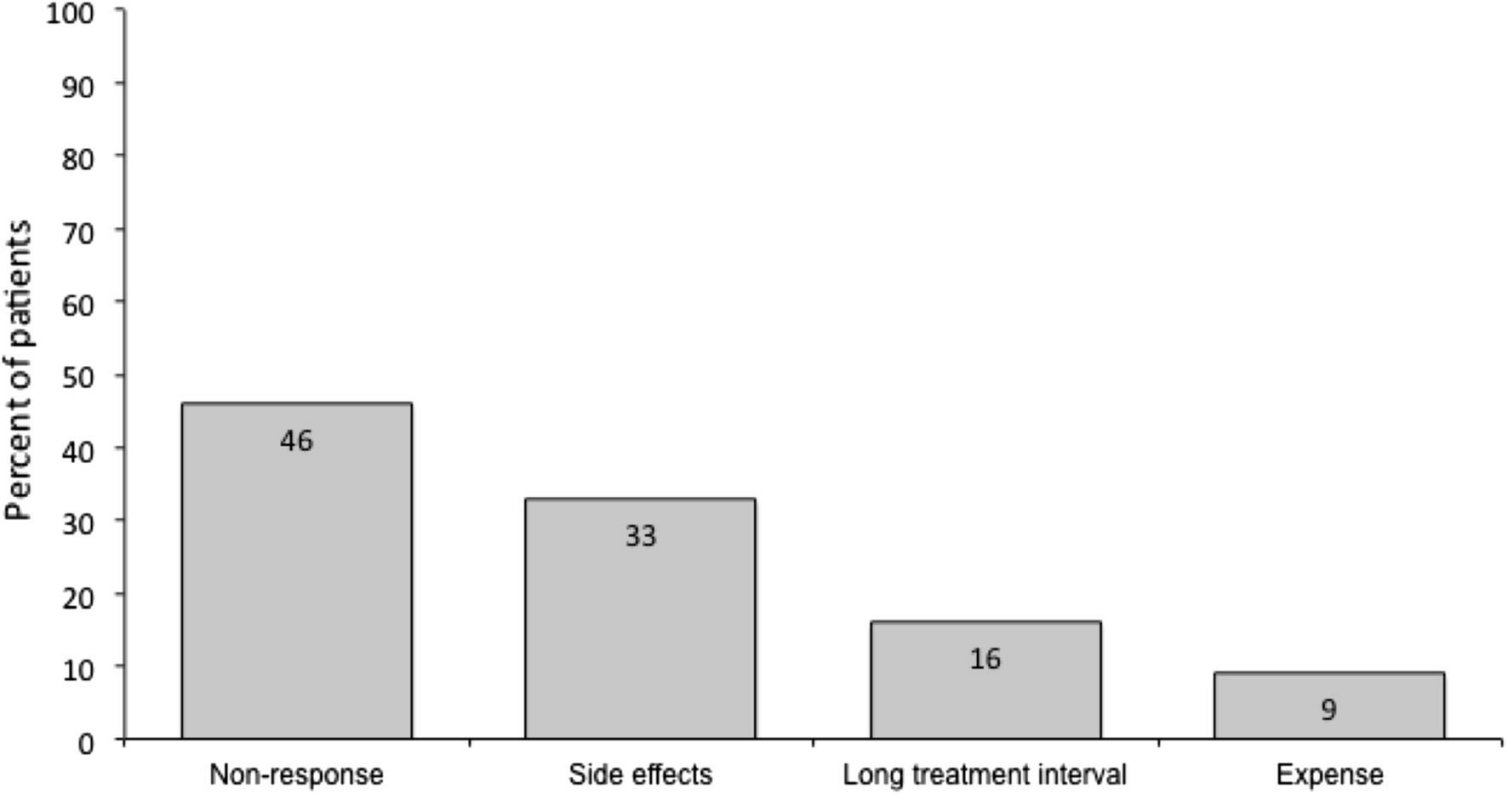
Reasons for not being satisfied with botulinum toxin. Base = 400 patients who did not report satisfaction with botulinum toxin treatment. Patients were asked: why were you not satisfied with your botulinum toxin treatment?

When patients who did not report satisfaction with BoNT treatment (including those who were either satisfied or dissatisfied; *n* = 400) were questioned further about their reasons, 46 % reported that BoNT A did not work for them and 33 % reported treatment side effects (Fig. 4). In addition, some patients reported that there was a long treatment interval (16 %) or that they had difficulty in attending regular appointments (3 %). When patients who had difficulty in attending regular appointments (*n* = 13) were questioned further about how long their treatments last, majority of these patients reported that their treatment lasts for ≤3 months.

Patients who did not receive treatment with BoNT (*n* = 128) cited dislike of toxins (20 %), expense (14 %) and dislike of injections (8 %) as key reasons for not having this treatment. In addition, 87 patients (68 %) cited ‘other’ reasons for not receiving BoNT. Although there was considerable variability in the verbatim texts, the most common ‘other’ reasons included lack of availability (many of these patients answered this question in the context of their first treatments and were now receiving BoNT), lack of physician awareness and presence of other dystonias.

## Discussion

Health-related quality of life has been defined as “the gap between our expectations of health and our experience of it” [16]. To the best of our knowledge, this International CD Patient Survey is the first and the largest survey undertaken to collect patient’s own perceptions of their CD management. In line with previous clinical studies, the survey found that the signs and symptoms of CD significantly impact on several aspects of daily life (including mood, sleep and pain [4, 17, 18]) and highlight the importance of managing the full range of problems a patient with CD might suffer.

Late diagnoses and misdiagnoses were a common complaint in this study. Although there are significant variations in the reported incidence of CD, one of the most recent studies conducted in the US reported a minimum incidence of 1.07 per 100,000 people [19]. This, however, is thought likely to be an underestimate, as community prevalence studies show that a significant population of people with CD do not seek treatment or are misdiagnosed [20]. Compounding the problem, significant variability in physicians’ ability to diagnose dystonia has also been reported, with studies showing that movement disorder specialists are more able to correctly diagnose CD than general neurologists [21]. It is hoped that the new definition and classification system for dystonia will help guide the diagnosis of this important disorder [22].

The study found that satisfaction with the information provided at diagnosis was generally low and the majority of patients used the internet to research their condition. Studies in neurology clinics indicate that dissatisfaction with information and emotional support provided by the physician may be important reasons for patients seeking a second opinion [23]. Interestingly, receiving a new diagnosis and/or treatment advice did not appear to influence satisfaction in this study. The high percentage of patients who used the internet to research their condition is likely due to the methodology used in this study in which patients were largely recruited through internet sites and social medial, biasing the study toward those patients with Internet access and skills.

From this study, patient expectations of treatment were high with almost two-thirds expecting to be ‘free’ of spasms or pain. These high expectations may have negatively influenced the satisfaction with overall (any) treatment of CD—whereas 64 % of patients reported symptom improvements with medical treatment, 49 % of patients were either equivocal or dissatisfied with their treatment outcome. This is an important insight, as treatment satisfaction has been shown to be a key factor in determining quality of life in CD [24]. The majority (86 %) of the patients were treated with BoNT therapy and thus the findings for patient satisfaction with BoNT were similar to satisfaction with treatment overall. Such findings highlight the need to explain to patients that while BoNT-A treatment is often effective, it is not a cure and there may be residual symptoms. Although patient satisfaction with BoNT treatment has not been found to correlate with the severity or complexity of CD [14], it is our experience (especially with more complex patients) that it is worth discussing that not all types of CD respond in the same way and there will be variability in how well patients respond to treatment [25].

It has been suggested that key steps in the management of CD involve the setting of realistic goals and prioritization of these goals with the patient [26]. When patients who were not satisfied with BoNT treatment (including the 20 % who were neither satisfied or dissatisfied) were questioned further about their reasons, most patients responded that BoNT “did not work” for them (46 % of reasons for non-satisfaction). The wording of this pre-defined response does not allow us to understand if all these patients truly had lack of efficacy, or if some other reason meant it did not work in the way the patients expected. We do not know in which settings (e.g., expert or community centers) the patients received treatment, and lack of efficacy can be due to poor injection technique (e.g., low doses or inappropriate muscle targeting). It may also be that the survey included a subgroup of very severely affected patients who might be candidates for deep brain stimulation (DBS). Indeed 9 % of patients in this survey reported previous surgery and or DBS. Other reasons cited included side effects (33 %), long treatment intervals or difficulties in attending appointments; the latter two reasons may be influenced by the healthcare systems of the respondents’ countries where the minimum interval between treatments may be different (longer) to the minimum interval of 3 months stipulated in the label of all BoNT products.

Limitations of this study are those inherent to patient surveys, which are based on the patient’s own understanding of their condition, and are not compared with objective clinical information (e.g., about disease severity, or response to treatment). A key limitation was the self-identification of the respondents as CD patients. There was no verification of diagnosis other than self-report, and the survey did not collect information regarding the type of CD suffered (e.g., predominant pattern or whether the dystonia was idiopathic, inherited or acquired). Overall, 37 % patients said they were affected by another movement disorder and 31 % patients said they had also had ‘other’ types of dystonia, but no further information on these important aspects was collected. Another limitation is the recruitment method in which only those patients with access and skills in internet use would be likely to respond to the survey and presumably only a percentage of patients who were invited to complete the survey through the media campaign did so. We do not know how many people received the invitation, and therefore cannot comment on this source of potential bias. Further, the use of this online tool meant that detailed analyses of answers was not feasible. Finally, there was an over-representation of responses from the US (*n* = 389), while 24 of the 38 individual countries had representation from <10 patients. In addition, when looking at the ‘other’ reasons for not having BoNT therapy, a number of patients responded that there had been a lack of availability of BoNT treatment in their area, but that they were now receiving it, thereby indicating that they had misunderstood the time frame of this question.

Despite the limitations in this type of study, this survey provided some interesting insights related to self-reported CD and its management. It highlights that diagnosis of CD is still delayed despite increasing awareness of the disorder. It suggests that the patients need to be better informed about their condition, the treatments available and the limitations of those treatments. It may be that realistically managing patient expectations of treatment would reduce the dissatisfaction of some patients.

## Appendix: International Cervical Dystonia Patient Survey

**Patient demographics**

D1. What is your gender?

- ○ Female
- ○ Male

D2. What is your age?

- ○ 18–24
- ○ 25–34
- ○ 35–44
- ○ 45–54
- ○ 55 and older

D3. In which country do you currently live?

- ○ Australia
- ○ Austria
- ○ Belgium
- ○ Canada
- ○ Croatia
- ○ Denmark
- ○ Finland
- ○ France
- ○ Germany
- ○ Ireland
- ○ Italy
- ○ The Netherlands
- ○ New Zealand
- ○ Norway
- ○ Poland
- ○ Portugal
- ○ Spain
- ○ Sweden
- ○ Switzerland
- ○ United Kingdom
- ○ United States
- ○ Other, please specify

D4. Which is your current work situation?

- ○ Full-time (more than 30 h a week)
- ○ Self-employed
- ○ Part-time
- ○ Full-time student
- ○ Retired
- ○ Not able to work/disabled
- ○ Not currently employed

**Patient background questions**

1. When were you diagnosed with cervical dystonia (also known as spasmodic torticollis)?
  - Less than a year ago
  - 1–5 years ago
  - 5–10 years ago
  - 10–15 years ago
  - More than 15 years ago
2. If you also have another type of dystonia, which do you have? **[SELECT ALL THAT APPLY]**
  - Blepharospasm
  - Hemifacial spasm
  - Other, please specify
  - I do not have another type of dystonia

**Change in quality of life**

3. After your first symptoms began, how did they affect and change your life? **[SELECT ALL THAT APPLY]**
  - Not able to work anymore
  - Loss of independence in everyday life activities (not able to drive, personal care)
  - Relationships with family members suffered
  - Not able to go shopping
  - No leisure activities (restaurants, theatres)
  - Depression and mood alterations
  - Experienced a lot of pain
  - Lack of sleep
  - Increase in alcohol intake
  - No effects or changes
4. After your first symptoms began, which of the following areas of life were affected when your symptoms were at their worst? **[SELECT ALL THAT APPLY]**
  - Family
  - Marriage/relationships
  - Health
  - Finances
  - Work/school
  - Well being
  - Other, please specify
  - No effect on relationships

**Unmet needs—patients without confirmed diagnosis and treatment started**

5. After your first symptoms began, when were you diagnosed with cervical dystonia?
  - 0–6 months later
  - 6–12 months later
  - More than 1 year later
  - More than 5 years later
6. Before your diagnosis of cervical dystonia, were you misdiagnosed with any of the following related neurological movement disorders? **[SELECT ALL THAT APPLY]**
  - Cervical muscle strain
  - Tremor
  - Chorea
  - Myoclonus
  - Tardive syndromes
  - Epilepsy
  - Cerebral palsy
  - Psychological illness/stress-related disorder
  - Others, please specify
  - No, I was never misdiagnosed
7. After you were diagnosed with cervical dystonia, where did you look for more information about your condition? **[SELECT ALL THAT APPLY]**
  - Internet
  - Patient association
  - Printed information in the doctor’s office
  - From general practitioner/family practice doctor
  - Other, please specify
  - Did not search for more information

**Patient’s expectations regarding relationship with doctors**

8. When you first talked to your neurologist/movement disorder specialist were you satisfied with the information you received about your condition?
  - Yes
  - No
9. **IF Q8** **=** **B**, why were you NOT satisfied with the information you received about your condition? **[SELECT ALL THAT APPLY]**
  - Not provided with enough information
  - My doctor used too many technical words
  - My doctor did not spend enough time to explain the diagnosis
  - I felt overwhelmed with information
  - Problems with communication due to language/dialect issues
  - Other, please specify
10. **IF Q9** **=** **C**, how much time with your neurologist/movement disorder specialist did you think was required when first discussing your diagnosis?
  - 20–30 minutes
  - 30–40 minutes
  - More than 40 minutes
11. Were you satisfied with the overall relationship you had with your doctor?
  - Yes
  - No
  - Not completely
12. **If Q11** **=** **B,C** why were you not completely satisfied with the overall relationship you had with your doctor?

**Overall Approach to treatment of Dystonia**

13. Did you discuss your expectations regarding treatment with your doctor?
  - Yes
  - No
14. If **Q13** **=** **A**, after discussing the different treatment options with your doctor, what were your expectations regarding your overall treatment plan? **[SELECT ALL THAT APPLY]**
  - To be free of spasms
  - To be free of pain
  - To feel well overall
  - To feel confident
  - To be able to work
  - To be able to sleep better
  - To be able to drive
  - To be able to have fun, enjoyment (leisure)
  - To return to normal routine before my symptoms began
15. If you were given support materials to read about cervical dystonia when you were diagnosed by your doctor or nurse, what were you given? **[SELECT ALL THAT APPLY]**
  - Leaflets and brochures
  - Patient organisations to contact
  - Support group information
  - Doctor found information on computer
  - Did not receive any support materials
16. What best describes your current healthcare finances? **[SELECT MOST SUITABLE OPTION]**
  - Government funded/government program
  - Private healthcare insurance
  - Combination of personal and government funded/government program
  - Public healthcare plan
  - Self-funded
17. Does your current healthcare insurance plan impact your treatment options?
  - Yes
  - No

**Practical treatments of Dystonia**

18. When you were diagnosed with cervical dystonia, which, if any, of the following practical improvements did you try? **[SELECT ALL THAT APPLY]?**
  - Using a foam/water pillow
  - Using a water bed
  - Massages (shiatsu, etc.)
  - Reflexology
  - Pilates/Tai Chi/Yoga
  - Creative or artistic activities (painting, singing, music therapy)
  - Gardening
  - Stress reduction training
  - Light exercise (walking, swimming, dancing)
  - Physiotherapy/physical therapist
  - General rest
  - None of these
19. Which, if any, of the following practical suggestions helped reduce your symptoms? **[SELECT ALL THAT APPLY]?**
  - Using a foam/water pillow
  - Using a water bed
  - Massages (shiatsu, etc.)
  - Reflexology
  - Pilates/Tai Chi/Yoga
  - Creative or artistic activities (painting, singing, music therapy)
  - Gardening
  - Stress reduction training
  - Light exercise (walking, swimming, dancing)
  - Physiotherapy/physical therapist
  - General rest
  - None of these
20. As a result of your practical improvements, which of the following areas did you feel most benefit? **[SELECT ALL THAT APPLY]**
  - Regained independence
  - Gained confidence
  - Able to play sports
  - Mood improvement
  - Able to go back to work
  - Less anxious driving
  - Developed new skills
  - Changes in relationships
  - Improvement of my symptoms
  - Other, please specify
  - No changes

**Medical Treatments of Dystonia**

21. Which treatment options did your doctor suggest to help your condition? **[SELECT ALL THAT APPLY]**
  - Oral medication
  - Botulinum toxin injections
  - Physiotherapy/physical therapist
  - Surgery/deep brain stimulation
  - Psychological support/counselling support
  - Other, please specify
  - None of these
22. Which treatment option did you choose or did your doctor prescribe? **[SELECT ALL THAT APPLY]**
  - Oral medication
  - Botulinum toxin injections
  - Physiotherapy/physical therapist
  - Surgery/deep brain stimulation
  - Psychological support/counselling support
  - Other
23. As a result of the treatment option you chose or was prescribed by your doctor, which of the following areas did you feel most benefit? **[SELECT ALL THAT APPLY]**
  - Regained independence
  - Gained confidence
  - Able to play sports
  - Mood improvement
  - Able to go back to work
  - Less anxious driving
  - Developed new skills
  - Changes in relationships
  - Improvement of my symptoms
  - Other, please specify
  - No changes
24. How satisfied are you with the treatment outcomes on your condition?
  - Completely satisfied
  - Fairly satisfied
  - Neither satisfied nor dissatisfied
  - Fairly dissatisfied
  - Completely dissatisfied

**Patient’s expectations of Botulinum Toxin (BoNT A)**

**Questions 25–30 are for respondents who selected option B in Q21**

25. Did you discuss the benefits and side effects of botulinum toxin with your doctor?
  - Yes
  - No
  - Not enough, please specify
26. Since receiving treatment with botulinum toxin have your symptoms:
  - Improved
  - Stayed the same
  - Worsened
27. Based on your experience with botulinum toxin treatment, how long do you have to wait between treatments?
  - 3 months
  - 4–6 months
  - 6 months to 1 year
  - Receive my botulinum toxin treatment when I need it
28. How do you rate the overall satisfaction of botulinum toxin treatment?
  - Completely satisfied
  - Fairly satisfied
  - Neither satisfied nor dissatisfied
  - Fairly dissatisfied
  - Completely dissatisfied
29. If **Q28** **=** **C,D,E,** why were you not satisfied with your botulinum toxin treatment? **[SELECT ALL THAT APPLY]**
  - It did not work for me
  - Treatment side effects
  - Too expensive
  - Treatment interval is too long
  - Too difficult to attend regular treatment
  - Other, please specify
30. If **Q29** **=** **D**, how long do the effects of your botulinum toxin treatment last?
  - 1–2 months
  - 2–3 months
  - 3–4 months
  - 4–5 months
  - 5–6 months
  - Over 6 months
31. If **Q22** **=** **C, **was it helpful to visit a physiotherapist who specialises in dystonia?
  - Yes
  - No
  - Did not visit a physiotherapist who specialises in dystonia
32. If **Q22** **=** **A,D,E,F,** for what reason(s) were you not prescribed botulinum toxin treatment? **[SELECT ALL THAT APPLY]**
  - Have a contraindication (e.g. pregnancy, allergy of botulinum toxin components etc.)
  - Inconvenience/trouble of returning for treatment
  - Do not like the idea of putting toxins into my body
  - Do not like injections
  - Too expensive
  - Other, please specify

**Personal experiences regarding patient care for dystonia**

33. Since you were diagnosed with cervical dystonia, did you contact a patient organisation or support group?
  - Yes
  - No
34. **If Q33** **=** **A**, how did you receive the details of the patient organisation or support group?** [CHOOSE ONE]**
  - Through the physician
  - Through the nurse
  - Leaflet/poster information in the hospital
  - Internet (search engine)
  - Social media (facebook, twitter)
35. **If Q33** **=** **A**, if you joined a dystonia patient organisation or support group, would you recommend that patient organisation or society to othercervical dystonia patients?
  - Yes
  - No
  - I didn’t join a patient group or society
36. **If Q35** **=** **B**, why would you NOT recommend the patient organisation or support group you joined? **[SELECT ALL THAT APPLY]**
  - They do not offer the help I need
  - The group is not interactive
  - They do not provide enough educational materials/information about my condition
  - Other, please specify
37. Did your doctor ask you to feedback after your treatment?
  - Yes
  - No
38. If **Q37** **=** **A**, how were you asked to provide this feedback?
  - Arrange another appointment
  - Write or email the doctor
  - Other, please specify
